# Trajectories of Health-Related Quality of Life and HbA1c Values of Children and Adolescents With Diabetes Mellitus Type 1 Over 6 Months: A Longitudinal Observational Study

**DOI:** 10.3389/fped.2019.00566

**Published:** 2020-01-21

**Authors:** Kathrin I. Fischer, Felix H. Fischer, Dana Barthel, Christiane Otto, Ute Thyen, Marcus Klein, Otto Walter, Ulrike Ravens-Sieberer, Matthias Rose, Sandra Nolte

**Affiliations:** ^1^Department of Psychosomatic Medicine, Center of Internal Medicine and Dermatology, Berlin Institute of Health, Charité – Universitätsmedizin Berlin, Corporate Member of Freie Universität Berlin, Humboldt-Universität zu Berlin, Berlin, Germany; ^2^Department of Child and Adolescent Psychiatry, Psychotherapy, and Psychosomatics, Center for Psychosocial Medicine, University Medical Center Hamburg-Eppendorf, Hamburg, Germany; ^3^Research Unit Child Public Health, Department of Child and Adolescent Psychiatry, Psychotherapy, and Psychosomatics, Center for Psychosocial Medicine, University Medical Center Hamburg-Eppendorf, Hamburg, Germany; ^4^Department of Pediatric and Adolescent Medicine, Universität zu Lübeck, Lübeck, Germany; ^5^Department of General Pediatrics, Christian-Albrechts-Universität, Kiel, Germany; ^6^Department of Quantitative Health Sciences, University of Massachusetts Medical School, Worcester, MA, United States; ^7^Public Health Innovation, Population Health Strategic Research Centre, School of Health and Social Development, Deakin University, Geelong, VIC, Australia

**Keywords:** health-related quality of life, pediatrics, self-report, patient outcome assessments, diabetes mellitus type 1, computer-adaptive testing

## Abstract

**Introduction:** To achieve optimized blood glucose concentrations (assessed by HbA1c) and high health-related quality of life (HRQL), children and adolescents with diabetes mellitus type 1 (T1DM) must follow strict disease management strategies. This study aims to investigate HRQL of children and adolescents with T1DM and its association with HbA1c values over the course of 6 months.

**Methods:** Patients aged 7–17 years (*n* = 203) with T1DM provided HRQL data on a monthly basis. HRQL was measured using the Kids-CAT, a computer-adaptive test (CAT) comprising five generic HRQL domains. HbA1c concentrations were assessed at baseline, at 3 and 6 months. We explored the trajectory of HRQL at the domain level using linear mixed effects models. Further, we investigated the association between HRQL and HbA1c concentrations over time using path analysis models.

**Results:** Children and adolescents with T1DM reported high scores across all HRQL domains over time. However, those with an HbA1c concentrations of >9.0% reported significantly lower scores in physical well-being and parent relations compared with those with an HbA1c concentration of <7.5%. Path analysis models revealed a minimal temporal relationship between HbA1c and HRQL, with a small negative impact of HbA1c on physical well-being, psychological well-being and parent relations.

**Conclusion:** Although observed HRQL of young patients with T1DM was comparable to age-related German-speaking reference population over the course of 6 months, those with an HbA1c concentration >9.0% reported lower scores in selected HRQL domains. Thus, special attention should be drawn to HRQL of children and adolescents with higher HbA1c concentrations. The minimal relationship between HbA1c and HRQL indicates that the two therapy goals, i.e., achievement and maintenance of glycemic targets and high HRQL, should be considered and evaluated independently in clinical routine.

**Trial Registration**: DRKS00006326 (German Clinical Trial Register), date of registration: August 1st, 2014.

## Introduction

Continuous development of treatment opportunities for diabetes mellitus Type 1 (T1DM) allows children and adolescents with T1DM to live a relatively normal life despite their chronic condition. However, the number of children and adolescents diagnosed with T1DM is increasing and all patients affected have to comply with lifelong treatment and care (1, 2). The main T1DM treatment aim is to achieve or maintain glycemic targets to avoid acute and long-term complications (3, 4). Hence, observation of HbA1c concentrations is a crucial indicator to monitor this primary treatment goal (5, 6). Besides the glycemic target, an overarching treatment goal in diabetes care is achieving and maintaining high quality of life, and in particular health-related quality of life (HRQL), of young patients (4, 5).

In terms of glycemic target, the American Diabetes Association recommends age-independent HbA1c concentrations of <7.5% in children and adolescents (5, 7). However, studies have shown that most children and adolescents with T1DM do not meet these recommendations despite comprehensive treatment methods and technological advancements (1, 4, 8, 9). Non-achievement of the glycemic target are due to multiple factors, such as age, sex and quality of life, especially emotional and psychosocial aspects (10–14).

While a precise glycemic target has been defined for children and adolescents with T1DM, it is not the case for the overarching treatment goal HRQL, a multidimensional construct containing physical, mental and social aspects of health (15). Various measures to assess generic as well as diabetes-specific HRQL are available (16, 17). Diabetes-specific HRQL measures assess disease-specific health problems and symptoms. The measurement of generic HRQL allows the comparison of results to healthy peers and the identification of potential problems, which go beyond diabetes-specific symptoms or treatment issues, but are related to metabolic control and diabetes management (16, 18). As outlined by Hilliard et al. (18), HRQL does not only function as a classical outcome parameter but might also serve as measure to identify problems in physical, emotional or social well-being, i.e., aspects that are related to diabetes management and consequently to the glycemic target (18).

So far, HRQL is usually not routinely assessed in clinical practice, but various studies investigated HRQL in children and adolescents with T1DM in comparison with healthy peers and also its association with other clinical parameters. Cross-sectional studies reported varying results when comparing HRQL of children with T1DM with healthy peers. While Varni et al. (19) found significantly lower scores in the domains emotional, psychosocial, and school functioning, Murillo et al. (14) found only slightly lower scores in the domain physical well-being, when comparing children with T1DM with healthy peers (14, 19). In contrast, Wagner et al. (20) reported no differences between children with T1DM with healthy peers, but higher psychological and school well-being scores in sub-groups of children with T1DM (20).

The relationship between the clinical outcome parameter HbA1c concentration, as an indicator for the glycemic target, and HRQL has also been investigated in young patients with diabetes mellitus. While some studies, found no associations between HRQL and glycemic target (21–23), others were able to detect a relationship between both the two outcome parameters (24–27). For example, it was found that higher scores in HRQL were associated with better metabolic control (25), while poor metabolic control was related to psychosocial problems in children and adolescents with T1DM (12, 27).

While most studies were based on cross-sectional data, less is known about the association of HRQL and HbA1c over time. To fill an important gap in the literature, this study aims to investigate the disease trajectory of children and adolescents with T1DM and in what way their self-reported HRQL is associated with HbA1c concentrations. Based on previous research, we hypothesized that high HbA1c concentrations are associated with lower HRQL over time.

## Materials and Methods

We followed the STROBE Statement und used the STROBE checklist for reporting results of this study (28, 29). The completed STROBE checklist can be found in Table S1.

### Study Design and Setting of the Study

The present study includes a subsample of children and adolescents with T1DM of the Kids-CAT project. The Kids-CAT project aimed to develop, validate and implement the first German-speaking computer-adaptive test (CAT) to measure HRQL in children and adolescents with chronic conditions (30–32). The prospective longitudinal observational study took place at two pediatric outpatient clinics (Kiel and Lübeck) at the University Medical Center Schleswig Holstein, Germany from June 2013 to October 2014. We applied a convenience sampling strategy, where study nurses at both clinics recruited children and adolescents with chronic conditions (asthma, juvenile arthritis, and diabetes mellitus) who attended the clinic for regular examination. Inclusion criteria were age between 7 and 17 years, clinical diagnosis of diabetes mellitus, asthma or juvenile arthritis and sufficient knowledge of German (spoken and written). The sample size of the study was determined according to the primary objective of the Kids-CAT project. For validation purposes, a sample size of 300 participants were required. A total of 312 children and adolescents participated in the Kids-CAT project, including 205 children and adolescents with diabetes mellitus.

The study was conducted adhering to the Declaration of Helsinki. Ethical approval was granted by Chambers of Physicians Kiel and Lübeck and the Chamber of Psychotherapists Hamburg, Germany. Written informed consent was obtained from parents/legal guardians, informed assent was obtained from children and adolescents.

### Participants and Procedure

This study reports data of a subsample including all children and adolescents diagnosed with diabetes mellitus [based on the International Statistical Classification of Disease and Related Health Problems, 10th revision, German Modification (ICD-10-GM) code E 10: Diabetes mellitus Type 1 (33)].

Assessment of HbA1c and HRQL took place at the respective outpatient department or at home. For the HRQL questionnaire, children and adolescents completed these electronically on a laptop, PC, tablet, or smartphone at the outpatient department during the waiting time (clinical assessment) or at home (home assessment). The clinical assessment was embedded in the routine medical encounter of the young patients (approximately once every 3 months). In addition to patient assessments, pediatricians provided further clinical information. For home assessments, study nurses sent a link via email to participants 1–2 days before the predetermined measurement point. Participants were reminded to complete the survey up to three times. For this study, data of seven measurement points over the course of 6 months with monthly intervals between measurements (*M* = 33.04 days; *SD* = 11.362) were analyzed.

### Measures and Instruments

We measured self-reported HRQL by use of the Kids-CAT. This generic HRQL instrument comprises the domains physical well-being, psychological well-being, parent relations, social support and peers, and school well-being akin to the KIDSCREEN domain structure with a recall period of 1 week (31, 34). The Kids-CAT was developed based on classical test theory and item response theory (IRT) (30, 35). For each of the five health domains an item bank was developed and calibrated including between 26 and 46 items. Thus, a latent trait level (T-score), including the standard error, can be estimated based on a single item. By applying additional items of the same the item bank, the latent trait level of the domain is re-estimated and the measurement precision increases. The Kids-CAT adapts to the individual, meaning that a patient has to answer only a subset of items of a domain, which are selected based on the patient's responses to the first item(s) (altogether three to seven items per domain). Although participants answer different subsets of questions, their estimated latent trait levels (T-scores) are comparable across individuals as well as within individuals over time. The estimated trait levels (higher score indicating better HRQL) for each domain are anchored to a German speaking reference sample (*n* = 10,577–19,580), meaning that a T-score of 50 with a SD ± 10 corresponds the average HRQL of children and adolescents in the German general population. Measurement properties of the Kids-CAT have been shown to be valid and reliable (31). Further information on the development process can be found elsewhere (30).

We assessed clinical data during the clinical measurement points (at three time points) based on both medical records and forms filled out by pediatricians. HbA1c concentrations for each clinical measurement point were grouped into <7.5, 7.5–9.0, and >9.0% (5, 36). In addition, we calculated the average HbA1c concentration over 6 month for each participant with at least two data points and grouped the average HbA1c concentration as described above. Further, we collected data on the type of therapy (categorical variable) with the options pump therapy, injection therapy, or switching therapy (from injection to pump therapy or vice versa within the period under review), age at the time of diagnosis (discrete variable), duration of the disease in years (discrete variable), as well additional chronic condition(s) (dichotomous variable). Socioeconomic status of the family was assessed based on nine validated questions on education (two items), income (three items), and occupation (four items) of the parents developed by the Robert Koch-Institute and used in German population-based studies (37, 38). We asked parents to complete these questions at baseline. Due to the responses, an SES index (3–21 points) was computed and categorized into high (13.9–21.0 points), middle (8.0–13.8 points), and low (3.0–7.9 points) social status.

### Statistical Analyses

First, we performed descriptive analyses at the group level to characterize the study sample and evaluate self-reported HRQL data according to the five Kids-CAT domains over the course of 6 months. Further, we investigated the change in HRQL domains over time on an individual patient level by calculating the percentages of children and adolescents whose T-scores improved and declined from one to the next measurement point. We defined change (improvement or decline) as a T-score of the individual lying outside of the confidence interval of the T-score of the preceding measurement point. For that reason, we calculated for each participant the respective confidence interval on a domain level for each time point, using point estimate and respective standard error provided by the Kids-CAT.

Following these descriptive analyses, we conducted missing data analysis. We checked, if data was missing completely at random (MCAR) using Little's MCAR test and checked patterns of missing data (39). We performed multiple imputation analysis creating *m* = 20 datasets, using the predictive mean matching method for numeric data, logistic regressions for binary data and proportional odds models for ordinal data (39–41) for further analysis. Sensitivity analysis were run to compare the original data set and the pooled results of the imputed data sets.

In a third step, we investigated the associations between baseline HbA1c concentration and Kids-CAT domains over time. We grouped participants based on their baseline HbA1c concentration according to the American Diabetes Association (5) into three groups (1: <7.5%; 2:7.5–9.0%, 3:>9.0%) (5, 6). We investigated the change in HRQL on the domain level over time using linear mixed-effect models fitted by restricted maximum likelihood (REML) estimation. We modeled the effect of the HbA1c group and time as well as an interaction term of both variables (as fixed effects) for each Kids-CAT domain controlling for age and sex; we further included a random intercept for each individual (as random effect).

Finally, we investigated the temporal relationship between the HbA1c concentration and the five Kids-CAT domains over time. We applied an exploratory approach to develop and fit a path model for each Kids-CAT domain including the HbA1c concentration and the respective Kids-CAT domain score (at baseline, after 3 months, after 6 months). We estimated the effect of the HbA1c concentration on the domain T-score and vice versa over time, while controlling for age and sex. Model fit was evaluated by χ^2^ value and its associated *p*-value, Tucker Lewis Index (TLI), comparative fit index (CFI), and root means square error of approximation (RMSEA). We applied the cut of values ≥0.95 for TLI and CFI and <0.10 for RMSEA, and evaluated the significance level of χ^2^ (42).

Statistical analyses were performed using IBM SPSS Statistics for Windows Version 22 (descriptive analyses) and R Statistics version 3.3.2 using the packages mice for multiple imputation (41) and nlme (43), lme4 (44), lmerTest (45), and MuMIn (46) for mixed model analyses. For path analysis we used the package lavaan and semTools (47, 48). A *p*-value of <.05 was considered as statistically significant.

## Results

### Sample Description and Descriptive Analyses

**Figure 1:**
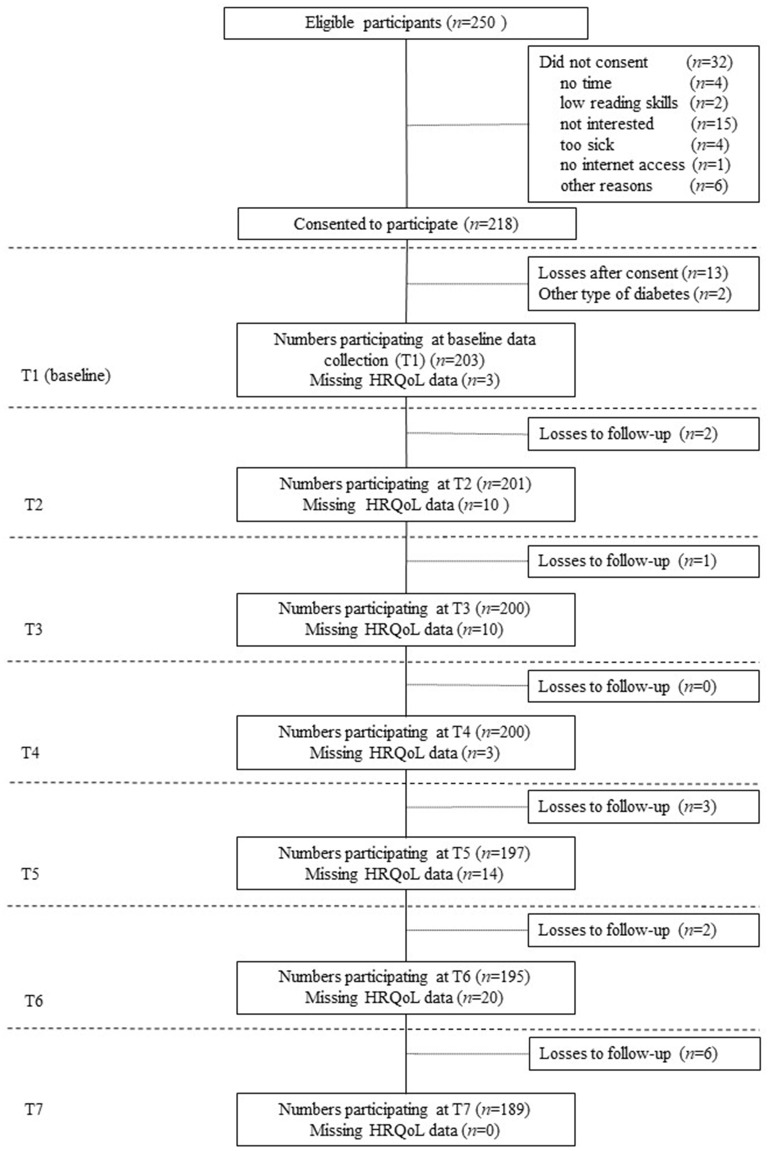
Flow diagram—study participation over time. Loss to follow-up was defined, as missing health-related quality of life (HRQL) data for all subsequent measurement points. Otherwise, missing HRQL data was considered as missing for the respective measurement point.

In total, 250 children and adolescents were eligible for study participation according to inclusion criteria. Of these, 218 consented for study participation, however 13 participants where lost after consensus and two participants were excluded from the analyses due to other type of diabetes mellitus. According to this, 203 children and adolescents were included in following analyses (Figure 1). There were no statistically significant differences in age (*M*_responder_ = 12.77, *SD* = 2.78; *M*_non−responder_ = 13.24, *SD* = 3.12; *p* = 0.295) and sex (female_responder_ = 44.3%; female_non_responder_ = 57.8%; *p* = 0.103) between young patients who participated in the study (responders) and those who did not participate (non-responders). Across the seven measurement points, 14 participants were lost to follow-up, which corresponds to 6.9% of the total sample. Participants were considered loss of follow-up, if no HRQL data was available for all subsequent measurement points, otherwise it was considered as missing data for the respective measurement point.

Mean age of the investigated children and adolescents was 12.77 years and 44.3% were female. Children and adolescents, who participated in our study had a mean disease duration of 5.36 years, and the mean age of primary manifestation of T1DM was 7.31 years. Further, co-morbidities were reported for 19.7% of children and adolescents. During the study, 29.7% of the children and adolescents were under insulin pen therapy, 58.9% used an insulin pump and 11.4% switched the type of therapy during the study. The mean NGSP HbA1c concentration at baseline was 7.97% (*SD* = 1.37, Range = 8.90) and slightly decreased after 6 months to 7.89% (*SD* = 1.38, Range = 9.40). Further information on sociodemographic and clinical characteristics are presented in Table 1.

**Table 1:** Sociodemographic and clinical characteristics of the investigated children and adolescents with T1DM [baseline assessment (T1)].

	***N***	**Mean (SD)**	**%**
**Sociodemographic characteristic**			
Age (in years)	203	12.77 (2.78)	
Age group			
Children (7–11 years)	73		36.0
Adolescents (12–17 years)	130		64.0
Sex (female)	90		44.3
Socioeconomic status of the family	166	13.44 (3.06)	
Low	5		3.0
Middle	115		69.3
High	46		27.7
**Clinical characteristics (physician reported)**			
Disease duration in years	203	5.36 (3.69)	
Age at disease onset	203	7.41 (3.82)	
Treatment	202		
Pen	60		29.7
Pump	119		58.9
Change of treatment	23		11.4
Co-morbidity (yes)	40		19.7
NGSP HbA1c concentration in %	196	7.97 (1.37)	
<7.5%	74		37.8
7.5–9.0%	88		44.9
>9.0%	34		17.3

**Figure 2:**
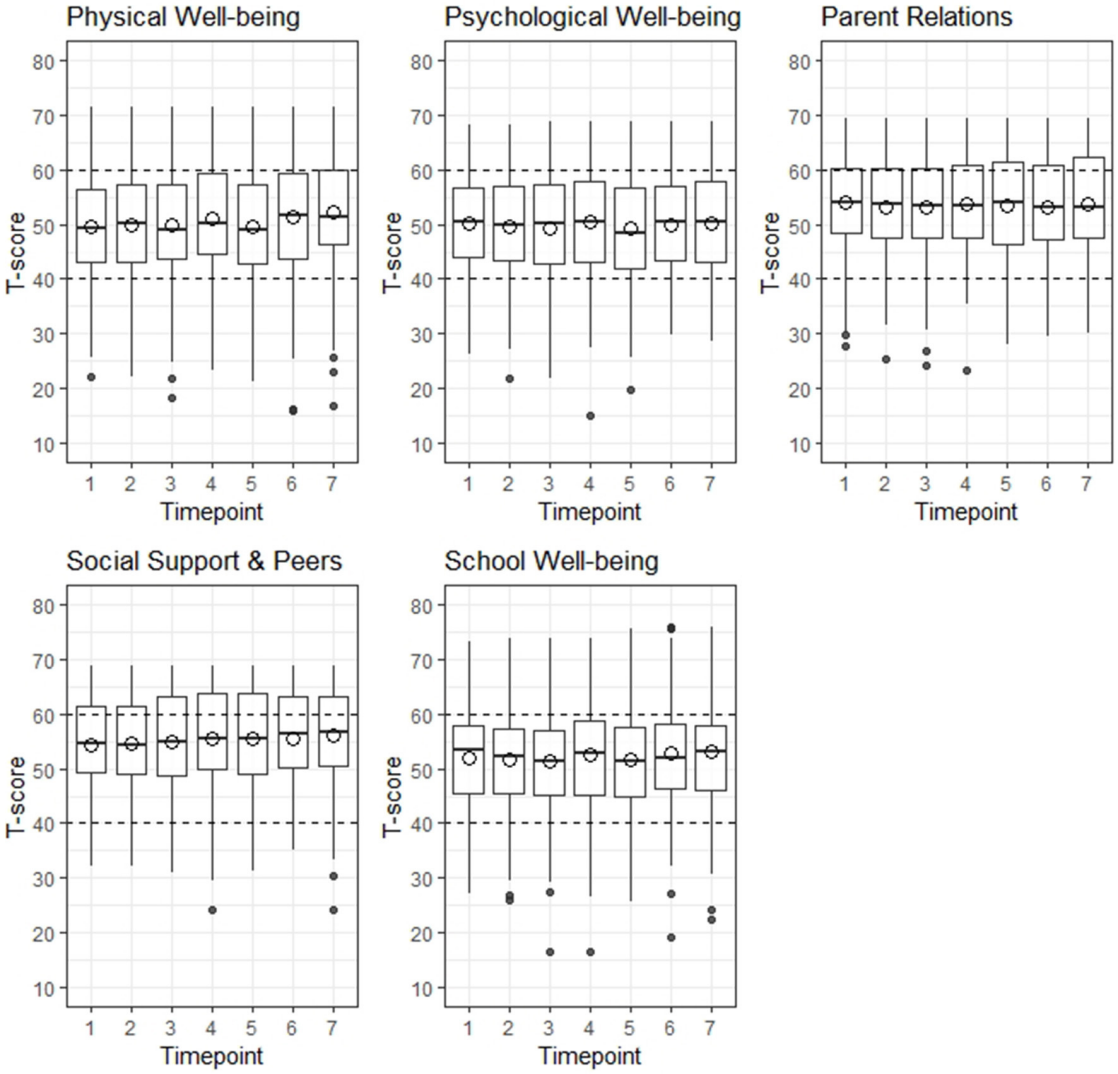
Distribution of health-related quality of life by Kids-CAT domain over 6 months. The area between the horizontal dashed lines indicate the normal range of a German age- and sex-matched norm population. The hollow circles depict the mean T-score. The black dots at the upper and lower end of the whisker indicate outliers.

Overall, T-scores for children and adolescents were comparable to the sex and age-matched German norm population for all five HRQL domains over the course of 6 month, meaning that mean T-scores were between 40 and 60 (Figure 2, detailed information on Kids-CAT T-scores on domain level over the course of 6 months are provided in Table S2). The range of T-scores was large in all five Kids-CAT domains, which was consistent over time. In addition, outliers were predominately found at the lower end of the range.

On an individual level, around 20% of the participants had significantly higher/lower T-scores from one to the following measurement point, which was consistent across all domains (average change by domain: physical well-being = 21.4%, psychological well-being = 22.9%, parent relations = 19.7%, social support & peers = 19.5%, school well-being = 19.7%) (detailed information are provided in Table S3).

In subgroup analyses, we investigated the change in HRQL (improvement or decline) from one to the following measurement point. If HRQL declined in children and adolescents from one measurement point to the following one, differences in T-scores were at least −7.53 in physical well-being (−6.51 for psychological well-being, −6.35 for parent relation, −6.49 for social support & peers, −6.45 for school well-being). Similar levels of difference scores were detected in those, who reported improvement in HRQL from one to the next measurement point (at least 7.15 for physical well-being, 6.73 for psychological well-being, 6.16 for parent relations, 6.26 for social support & peers, and 5.91 for school well-being were found; for detailed information see Table S4).

### Missing Data and Multiple Imputation

Analysis of missing data revealed that 10.3% of data was missing within our data set, varying by variable between 0 and 18.2%. Results of Little's MCAR test revealed that data are not completely missing at random (*p* < 0.01). Thus, we investigated patterns of missing data by visualization. We came to the conclusion that our data is at least missing at random, as no pattern of missing data could be found. Thus, we were able to use multiple imputation techniques to handle missing data. We created 20 datasets, and used these for the subsequent analysis (pooled data analyses).

### Trajectory of HRQL Over Time by HbA1c Categories

**Figure 3:**
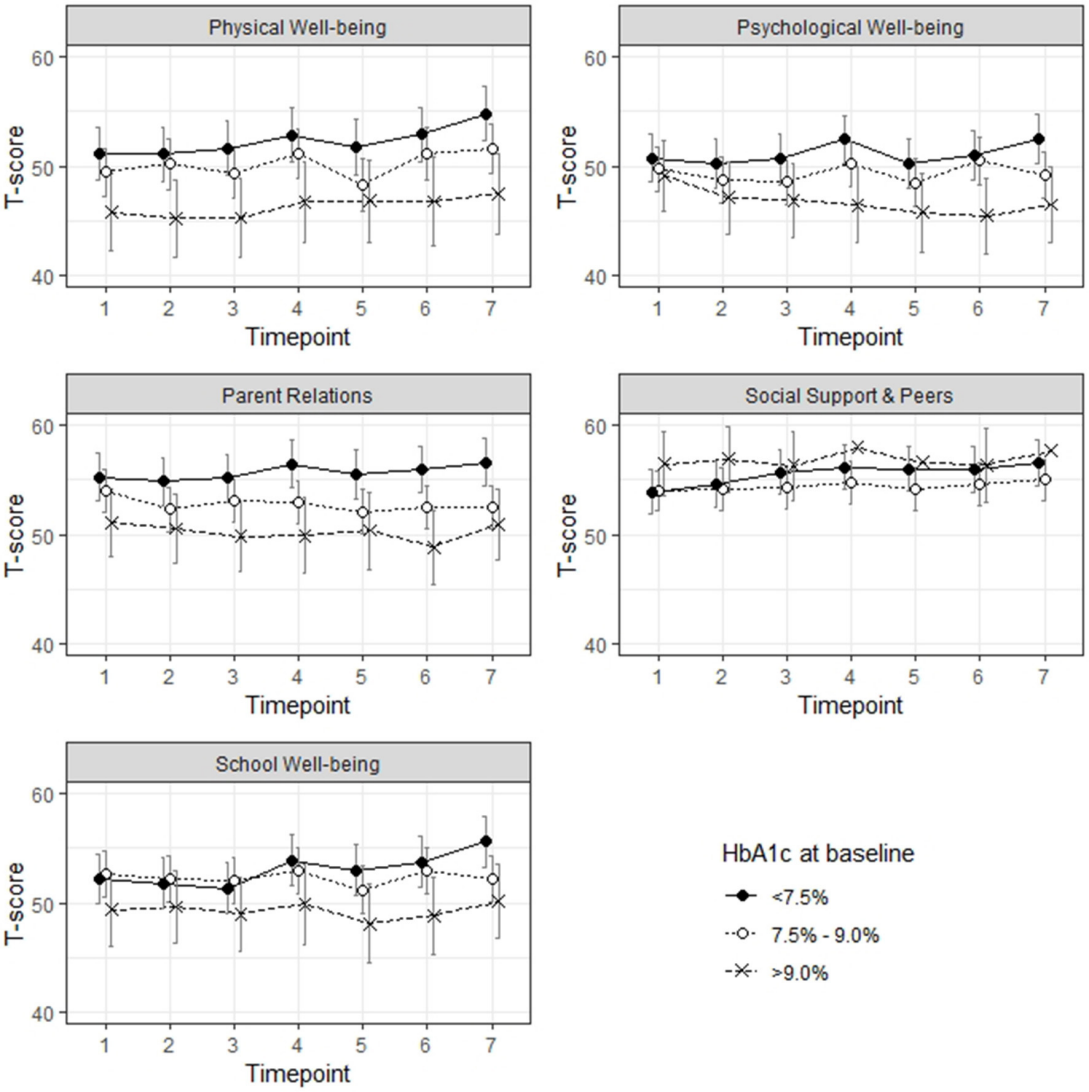
Health-related quality of life over the course of 6 months, group allocation according to respective HbA1c concentration at baseline.

In a next step, we were interested in the relationship between the three groups based on baseline HbA1c concentrations and HRQL measured by the Kids-CAT over time (Figure 3). Model estimates revealed that the group of children and adolescents with HbA1c concentrations >9.0% reported significant lower scores for the domains physical well-being (B = −5.60, *SE* = 2.21; *p* < 0.05) and parent relations (B = −4.32, *SE* = 1.99; *p* < 0.05) compared to the reference group (children and adolescents with a HbA1c concentration <7.5%). Nearly all models revealed only minimal change over time, only in the domains physical well-being, social support and school well-being statistically significant changes for a few selected time points were found. However, the change was less than ±4 points compared to the baseline value of these domains. For the domain psychological well-being, change over time based on the HbA1c groups indicated that those children with a baseline HbA1c concentration over 9.0% got worse over time, however, these findings were not statistical significant (results not shown).

### Association Between HbA1c Value and HRQL Over Time

**Figure 4:**
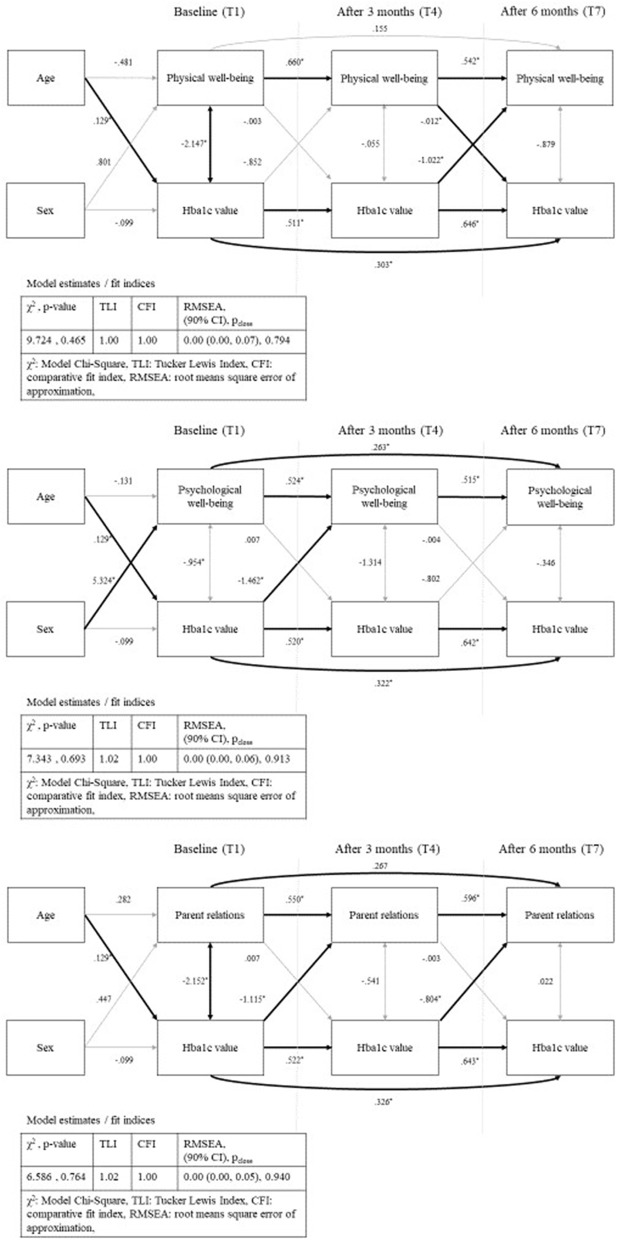
Path analysis—physical well-being, psychological well-being and parent relations with standardized parameter estimates and model fit indices; bold arrows show statistical significant paths (*p* < 0.05); sex as dichotomous variable (male).

In the path analyses, we modeled the effects of Kids-CAT domains (individual path model for each domain) and HbA1c over time [baseline (T1), after three (T4), and after 6 months (T7)] controlling for age and sex. In the first path, we estimated the direct effect of the respective domain over time (T1, T4, T7). The second path estimated the association of HbA1c concentrations over time (T1, T4, T7). Further, we modeled the effect of the baseline T-scores on the HbA1c concentration after 3 months, as well as the effect of the baseline HbA1c concentration on the T-scores after 3 months. Corresponding paths were specified from the respective scores measured at baseline as well as from those assessed after 3 months on scores after 6 months. In addition, correlations between HbA1c concentration and domain-specific T-scores (same measurement point) were estimated. Finally, we added the covariates age and sex to estimate the direct effect on baseline values. The final models with unstandardized parameter estimates for physical well-being, psychological well-being and parent relations are displayed in Figure 4 (path models for social support & peers and school well-being can be found in Figure S1). Fit indices of all five models showed good fit of the data according to TLI, CFI, and RMSEA, while χ^2^ was acceptable.

Estimates of all path analysis models showed statistically significant associations between the domain scores over time as well as between the HbA1c concentrations over time with nearer time points more strongly correlated. Further, we found statically significant positive associations between age and HbA1c concentration for the five models, but no statistically significant association between age and the five Kids-CAT domains. Statistical significant associations between sex and HRQL was only found for the domain psychological well-being.

The path analysis model for physical well-being and HbA1c concentrations showed negative correlations at baseline (B = −2.15, *SE* = 0.99, *p* < 0.05) that indicated that a higher HbA1c concentration was associated with lower physical well-being. For the temporal correlation, the model revealed statistically significant negative association between HbA1c concentration after 3 months and physical well-being after 6 months (B = −1.02, *SE* = 0.49, *p* < 0.05). Further, the model showed statistically significant negative associations between the path physical well-being after 3 months and HbA1c concentration after 6 months (B = −0.012, *SE* = 0.01, *p* < 0.05) (Figure 4). Remaining paths of the model were not statistically significant.

The path analysis model for psychological well-being and HbA1c concentrations revealed a statistically significant negative association between HbA1c concentration at baseline and psychological well-being after 3 months (B = −1.46, *SE* = 0.49, *p* < 0.05), which was not replicable for the path HbA1c concentration after 3 months and psychological well-being after 6 months (B = −0.80, *SE* = 0.47, *p* < 0.09). Other paths with regard to the association between HbA1c concentration and psychological well-being were not statistically significant.

The path analysis model for parent relations and HbA1c concentration showed statistically significant negative correlation between baseline HbA1c concentration and parent relations at baseline (B = −2.15, *SE* = 0.97, *p* < 0.05). Further, the model revealed a temporal relationship between HbA1c concentration and parent relations. Statistically significant negative associations between baseline HbA1c concentration and parent relations after 3 months (B = −1.12, *SE* = 0.41, *p* < 0.05), and HbA1c concentration after 3 months and parent relations after 6 months (B = −0.80, *SE* = 0.40, *p* < 0.05) were found. All remaining paths of the model were not statistically significant.

The path analysis models for social support & peers and school well-being revealed no statistical significant associations between the domain scores and HbA1c concentration (see Figure S1).

## Discussion

In the context of the Kids-CAT study, we explored associations between HbA1c concentrations and HRQL in children and adolescents with T1DM in continuous outpatient treatment over the course of 6 months. Overall, young patients reported average HRQL scores over a 6 months period. Further, for four out of five Kids-CAT domains we found statistically significant higher scores in young patients with a baseline HbA1c concentration <7.5% compared to those with a baseline concentration of >9.0%. At the group level, no significant change over time was detected. However, at the individual patient level, 20% of young patients reported change from one to the following measurement point in at least one HRQL domain. Exploring the temporal relationship between HbA1c concentration and the five Kids-CAT domains, only small associations were detected for the Kids-CAT domains physical and psychological well-being as well as parent relations.

The health-related quality of life scores of the investigated young patients with T1DM corresponded to an age and sex-matched German reference population (*M* = 50; *SD* = 10) (30). Similar findings were reported by previous studies for children and adolescents with T1DM who reported HRQL scores similar to their healthy peers (14, 19, 20). This finding was not surprising, considering the setting and the sample of our study. Assessments were completed during regular check-ups or at home by young patients living with T1DM on average for more than 5 years (range between 0 and 14 years). Thus, it can be assumed that diabetes management was well-adjusted and most participants were in stable health situations, where disease-related symptoms and the management of T1DM was only minimally affecting their generic HRQL (49, 50). However, further studies are needed to investigate the effect of the prolonged T1DM remission phase on HRQL.

At a group level, the changes in the five Kids-CAT domains over time were only minimal, which was expected given the observational study design. However, looking at an individual level, scores varied over time. One fifth of the young patients reported relevant change in at least one HRQL domain. Out of those, approximately 10% reported worse scores and 10% reported better scores from one to the following measurement point. The difference from one to the following measurement point was at minimum six points on the T-metric, which is statistically significant, and can be considered clinically meaningful (51). These findings indicate that regular monitoring of HRQL in clinical practice is useful to detect change early. Further exploration of predictors related to improvement of individuals over time could help to improve resilience and could facilitate to develop measures to improve HRQL of children with T1DM. Previous research found that unhealthy lifestyle habits, such as diet and low physical activity, are associated with lower HRQL (52). Promoting healthy life style habits in children with T1DM could be an important aspect to improve HRQL over time.

Only minimal changes within the tree groups (baseline HbA1c concentration <7.5%, 7.5–9.0%, >9.0%) were found in each of the five HRQL domains over time. However, statistical significant differences were found between those children with a baseline HbA1c concentration <7.5% and those children with a baseline concentration >9.0% in the domains physical well-being and parent relations, which corresponds to the findings of previous studies (26, 53). Given the increasing risk of diabetes-related late complications due to continuously high HbA1c concentration, the difference in HRQL between those young patients with a high and a low HbA1c concentration might increase over future years. Considering that we used a more liberal HbA1c target of 7.5% for this study (5), and not the lower HbA1c target of 7.0% recommended by the International Society for Pediatric and Adolescent Diabetes (ISPAD) or even the HbA1c target of 6.5% recommended by the National Institute for Health and Care Excellence (NICE), the differences between groups might be larger (6).

Previous studies showed that HRQL in patients with T1DM decreased with increasing symptoms and complications (54–56). Further, our results showed that psychological well-being of young patients with a baseline HbA1c concentration >9.0% decreased minimally over the course of 6 months. Given the enhanced risk of young patients with T1DM for developing depressive symptoms, special attention should be paid to the HRQL of children with high HbA1c concentrations (57, 58). We explored the association between HRQL and HbA1c concentration over time in terms of a temporal relationship controlling for age and sex. In contrast to Naughton et al. (59), we were not able to show a statically significant associations between age and HRQL and only statically significant associations between sex and psychological well-being. (59).

The pathway models revealed some statistically significant pathways between HRQL domains and HbA1c concentrations. However, we were not able to determine the direction of associations over time. Whereas, Naughton et.al. (59) reported negative associations between HbA1c and HRQL over time (59), we only found small associations and a tendency that higher HbA1c concentrations may lead to lower scores in the domains physical and psychological well-being as well as parent relations. Hesketh et al. (60) reported similar results for psychological well-being that they could not predict follow-up HbA1c concentrations based on domain scores. However, changes in HbA1c concentrations could be predicted by self-reported physical functioning (60). Our findings can be discussed with regard to the conceptual model by Wilson and Cleary (61) explaining the relationship between clinical variables and HRQL (61). A high HbA1c concentration, as a measure of the average blood sugar levels over time, does only minimally impact the symptom and functional status of the young patient. Further, similar associations between metabolic control, including aspects of diabetes treatment and medical adherence, and quality of life have been described (13, 14). These associations point out the complexity of diabetes management, with different factors being mutually dependent (3, 5). Thus, both treatment goals, low HbA1c concentrations and high HRQL have to be monitored and addressed individually in medical care.

### Limitations

This study has some limitations, which have to be discussed in relation to the findings. Not uncommon for a longitudinal study including several time points, we were facing over 10% of missing data. As missing data could affect the results and hence the interpretation of the results, we used state-of-the-art imputation techniques to handle the missing data in our data set. We conducted sensitivity analysis comparing results of the original data set and the pooled results of the imputed data sets.

Further, sample size calculation was based on validation purposes of the Kids-CAT and thus not powered for the analysis presented in the paper. We examined *post hoc* power to detect group differences between young patients with an HbA1c concentration of <7.5% and those with an HbA1c concentration of >9.0%. Considering a difference of five points as clinical relevant, the power of the study is slightly underpowered. For path analysis, our sample size complies only with the minimal requirements. Our ratio of 14.5 participants to one parameter estimated, corresponds to the general consensus of 10:1 ratio in structure equation modeling (62, 63). Thus, our sample size was sufficient for the explorative type of our analyses, but results should be interpreted correspondingly cautious. Owing to our sample size, setting and explorative nature of this study, generalizability of our results is limited. Replication of our results is needed including a bigger sample. In addition, the follow-up of only 6 months is a rather short period to evaluate HRQL considering the lifelong chronic disease.

Finally, due to the study design, we were not able to control for differences between home and clinical assessments. Considering the use of mHealth technology, allowing to complete HRQL assessments everywhere, future studies are needed to determine the potential effect of the setting on HRQL scores.

## Conclusion

Overall, children and adolescents with T1DM reported high scores in all five Kids-CAT domains over the course of 6 months referring to good HRQL. From one assessment to the next, 20% of the young patients reported clinically meaningful changes in their HRQL as measured with the Kids-CAT despite the observational study design. Young patients with high HbA1c concentrations reported significantly lower scores in the HRQL domains physical well-being and parent relations over the course of 6 months. Moreover, psychological well-being of young patients with high HbA1c concentrations deteriorated over time. Finally, only minimal associations between HbA1c concentrations and HRQL domains were found over the course of 6 month.

Finding of this study indicate that regular monitoring is not only needed for HbA1c concentrations, but also for HRQL in clinical practice. Both outcomes have to be measured in order to address and achieve the treatment goals of health care of young T1DM patients that are achieving the glycemic target as well as high HRQL. Hence, the implementation of measures to assess HRQL facilitates patient-centered care by providing important information to health care professionals.

## Data Availability Statement

The raw data supporting the conclusions of this article will be made available by the corresponding author to any qualified researcher on reasonable request.

## Ethics Statement

The studies involving human participants were reviewed and approved by Chamber of Physicians Kiel, Germany, Chamber of Physicians Lübeck, Germany, and Chamber of Psychotherapists Hamburg, Germany. Written informed consent to participate in this study was provided by the participants' legal guardian/next of kin.

## Author Contributions

UR-S, MR, CO, UT, and MK designed the study concept and design. OW, SN, CO, MR, and UR-S developed the Kids-CAT tool. DB, SN, UT, and MK supervised and managed the data collection. OW, CO, and DB were responsible for data surveillance and data quality and managed the data preparation. KF, FF, and SN conceptualized the paper and interpreted the data. FF supervised the statistical analysis performed by KF. KF wrote the first draft of the manuscript. All authors critically revised the manuscript, gave final approval for submitting the manuscript for review, and agreed to be accountable for all aspects of the work.

### Conflict of Interest

The authors declare that the research was conducted in the absence of any commercial or financial relationships that could be construed as a potential conflict of interest.

## Abbreviations

CAT: computer-adaptive test
IRT: Item response theory
T1DM: Diabetes mellitus type 1
HRQL: health-related quality of life.
