# Supplementary material for: Trajectories of Health-Related Quality of Life and HbA1c Values of Children and Adolescents With Diabetes Mellitus Type 1 Over 6 Months: A Longitudinal Observational Study
Source: Front Pediatr. 2020 Jan 21;7:566. doi: 10.3389/fped.2019.00566 (PMC6986264; doi:10.3389/fped.2019.00566)
Supplement: Supplementary file 1 [file Data_Sheet_1.docx]

**Supplementary material - Tables**

| **Table S1:** STROBE Statement—checklist of items that should be included in reports of observational studies | | | | |
| --- | --- | --- | --- | --- |
|  | Item No | Recommendation | Page  No | Relevant text passages from the manuscript |
| **Title and abstract** | 1 | (*a*) Indicate the study’s design with a commonly used term in the title or the abstract | 1,2 | Title and Abstract |
|  |  | (*b*) Provide in the abstract an informative and balanced summary of what was done and what was found | 2-3 | Abstract incl. background, methods, results and conclusion |
| Introduction | | | | |
| Background/rationale | 2 | Explain the scientific background and rationale for the investigation being reported | 4,5 |  |
| Objectives | 3 | State specific objectives, including any pre-specified hypotheses | 5 | Aim of the study including pre-specified hypothesis |
| Methods | | | | |
| Study design | 4 | Present key elements of study design early in the paper | 6 | Sub-heading “study design and setting of the study” |
| Setting | 5 | Describe the setting, locations, and relevant dates, including periods of recruitment, exposure, follow-up, and data collection | 6,7 | Sub-headings “study design and setting of the study” and “Participants and procedure |
| Participants | 6 | (*a*) *Cohort study*—Give the eligibility criteria, and the sources and methods of selection of participants. Describe methods of follow-up  *Case-control study*—Give the eligibility criteria, and the sources and methods of case ascertainment and control selection. Give the rationale for the choice of cases and controls  *Cross-sectional study*—Give the eligibility criteria, and the sources and methods of selection of participants | 6,7 | Sub-headings “study design and setting of the study” and “Participants and procedure |
|  |  | (*b*) *Cohort study*—For matched studies, give matching criteria and number of exposed and unexposed  *Case-control study*—For matched studies, give matching criteria and the number of controls per case |  | n.a. |
| Variables | 7 | Clearly define all outcomes, exposures, predictors, potential confounders, and effect modifiers. Give diagnostic criteria, if applicable | 7,8 | Sub-heading “Measures and Instruments” |
| Data sources/ measurement | 8* | For each variable of interest, give sources of data and details of methods of assessment (measurement). Describe comparability of assessment methods if there is more than one group | 7,8 | Sub-heading “Measures and Instruments” |
| Bias | 9 | Describe any efforts to address potential sources of bias | 9 | Sub-heading “Statistical analyses” |
| Study size | 10 | Explain how the study size was arrived at | 6 | “The sample size of this study was determined according to the primary objective of the Kids-CAT project. For validation purposes, a total sample size of n=300 participants were required. This study refers to the subsample of children and adolescents with T1DM (n=203).” |
| Quantitative variables | 11 | Explain how quantitative variables were handled in the analyses. If applicable, describe which groupings were chosen and why | 7,8 | Sub-heading “Measures and Instruments” |
| Statistical methods | 12 | (*a*) Describe all statistical methods, including those used to control for confounding | 9 | Sub-heading “Statistical analyses” |
|  |  | (*b*) Describe any methods used to examine subgroups and interactions | 9 | Sub-heading “Statistical analyses” |
|  |  | (*c*) Explain how missing data were addressed | 9 | Missing data analysis & multiple imputations |
|  |  | (*d*) *Cohort study*—If applicable, explain how loss to follow-up was addressed  *Case-control study*—If applicable, explain how matching of cases and controls was addressed  *Cross-sectional study*—If applicable, describe analytical methods taking account of sampling strategy | 9 | Sub-heading “Statistical analyses” |
|  |  | (*e*) Describe any sensitivity analyses |  | n.a. |

| Results | | | | |
| --- | --- | --- | --- | --- |
| Participants | 13* | (a) Report numbers of individuals at each stage of study—e.g. numbers potentially eligible, examined for eligibility, confirmed eligible, included in the study, completing follow-up, and analyzed | 10,11 Figure1 | Sub-heading “Sample description and descriptive analyses” |
|  |  | (b) Give reasons for non-participation at each stage | 10,11 Figure 1 | Sub-heading “Sample description and descriptive analyses” |
|  |  | (c) Consider use of a flow diagram | Figure 1 |  |
| Descriptive data | 14* | (a) Give characteristics of study participants (e.g. demographic, clinical, social) and information on exposures and potential confounders | 10,11, Table 1 | Sub-heading “Sample description and descriptive analyses” and Table 1 |
|  |  | (b) Indicate number of participants with missing data for each variable of interest | 12,13 | Sub-heading “ Missing data and multiple imputations |
|  |  | (c) *Cohort study*—Summarize follow-up time (e.g., average and total amount) | 6 | Methods, sub-heading “Study design and setting of the study” |
| Outcome data | 15* | *Cohort study*—Report numbers of outcome events or summary measures over time | 11-12*;* 13-15 | Outcome HRQoL and HbA1c value |
|  |  | *Case-control study—*Report numbers in each exposure category, or summary measures of exposure |  | *n.a.* |
|  |  | *Cross-sectional study—*Report numbers of outcome events or summary measures |  | *n.a.* |
| Main results | 16 | (*a*) Give unadjusted estimates and, if applicable, confounder-adjusted estimates and their precision (e.g., 95% confidence interval). Make clear which confounders were adjusted for and why they were included | 13-15 | Sub-heading “Trajectory of HRQoL over time by HbA1c categories” and “Association between HbA1c value and HRQoL over time” |
|  |  | (*b*) Report category boundaries when continuous variables were categorized |  | n.a. |
|  |  | (*c*) If relevant, consider translating estimates of relative risk into absolute risk for a meaningful time period |  | n.a. |
| Other analyses | 17 | Report other analyses done—e.g. analyses of subgroups and interactions, and sensitivity analyses |  | n.a. |
| Discussion | | | | |
| Key results | 18 | Summarize key results with reference to study objectives | 15-16 |  |
| Limitations | 19 | Discuss limitations of the study, taking into account sources of potential bias or imprecision. Discuss both direction and magnitude of any potential bias | 18 | Sub-heading “Limitations” |
| Interpretation | 20 | Give a cautious overall interpretation of results considering objectives, limitations, multiplicity of analyses, results from similar studies, and other relevant evidence | 19 | Sub-heading “Conclusions” |
| Generalizability | 21 | Discuss the generalizability (external validity) of the study results | 18 | Sub-heading “Limitations” |
| Other information | | | | |
| Funding | 22 | Give the source of funding and the role of the funders for the present study and, if applicable, for the original study on which the present article is based | 2 | Acknowledgements |

*Give information separately for cases and controls in case-control studies and, if applicable, for exposed and unexposed groups in cohort and cross-sectional studies.

**Note:** An Explanation and Elaboration article discusses each checklist item and gives methodological background and published examples of transparent reporting. The STROBE checklist is best used in conjunction with this article (freely available on the Web sites of PLoS Medicine at http://www.plosmedicine.org/, Annals of Internal Medicine at http://www.annals.org/, and Epidemiology at http://www.epidem.com/). Information on the STROBE Initiative is available at www.strobe-statement.org

| **Table S2:** Kids-CAT T-scores and 95% confidence intervals (95% CI) over the course of six months | | | | | | | | | | | |
| --- | --- | --- | --- | --- | --- | --- | --- | --- | --- | --- | --- |
| Dimension | | Physical well-being | | Psychological well-being | | Parent relations | | Social support & friends | | School well-being | |
| Time point | N | T-score (mean) | 95% CI | T-score (mean) | 95% CI  (mean) | T-score (mean) | 95% CI  (mean) | T-score (mean) | 95% CI  (mean) | T-score (mean) | 95% CI  (mean) |
| Baseline (T1) | 200 | 49.47 | 48.14 – 50.80 | 50.16 | 48.88 – 51.44 | 53.88 | 52.61 –55.15 | 54.34 | 53.17 – 55.51 | 51.93 | 50.54 – 53.31 |
| T2 | 191 | 49.86 | 48.33 – 51.40 | 49.49 | 48.12 – 50.86 | 53.16 | 51.80 – 54.53 | 54.74 | 53.51 – 55.98 | 51.68 | 50.27 – 53.10 |
| T3 | 190 | 49.81 | 48.25 – 51.38 | 49.42 | 47.95 – 50.90 | 53.14 | 51.78 – 54.50 | 55.03 | 53.68 – 56.38 | 51.25 | 49.87 – 52.64 |
| T4 | 197 | 50.96 | 49.42 – 52.51 | 50.48 | 48.96 – 52.00 | 53.81 | 52.49 – 55.13 | 55.50 | 54.08 – 56.91 | 52.49 | 50.90 – 54.08 |
| T5 | 183 | 49.60 | 47.92 – 52.51 | 49.24 | 47.68 – 50.80 | 53.34 | 51.82 – 54.87 | 55.62 | 54.17 – 57.08 | 51.76 | 50.90 – 54.08 |
| T6 | 175 | 51.39 | 49.65 – 53.14 | 50.03 | 48.54 – 51.52 | 53.17 | 51.69 – 54.65 | 55.55 | 54.20 – 56.91 | 52.79 | 51.26 – 54.33 |
| T7 | 189 | 52.23 | 52.68 – 53.78 | 50.20 | 46.68 – 51.71 | 53.63 | 52.18 – 55.07 | 56.07 | 54.71 – 57.42 | 53.07 | 51.61 – 54.54 |

| **Table S3:** Percentage of change in the five Kids-CAT domains over time | | | | | | | | | | |
| --- | --- | --- | --- | --- | --- | --- | --- | --- | --- | --- |
| Kids-CAT domain | Physical well-being | | Psychological well-being | | Parent relations | | Social support & peers | | School well-being | |
|  | ↑ (%) | ↓ (%) | ↑ (%) | ↓ (%) | ↑ (%) | ↓ (%) | ↑ (%) | ↓ (%) | ↑ (%) | ↓ (%) |
| T1 to T2 | 10.50 | 10.50 | 8.33 | 13.33 | 10.06 | 10.06 | 15.64 | 7.26 | 12.22 | 11.67 |
| T2 to T3 | 10.59 | 11.76 | 11.31 | 9.52 | 11.90 | 7.74 | 9.04 | 9.04 | 9.64 | 7.23 |
| T3 to T4 | 14.81 | 4.32 | 16.25 | 6.86 | 11.80 | 6.21 | 13.29 | 5.06 | 15.17 | 7.10 |
| T4 to T5 | 8.61 | 14.57 | 7.33 | 13.33 | 10.00 | 10.66 | 9.93 | 8.61 | 8.72 | 12.75 |
| T5 to T6 | 15.44 | 8.05 | 16.22 | 10.14 | 12.33 | 8.22 | 11.56 | 8.16 | 12.16 | 2.70 |
| T6 to T7 | 12.74 | 6.36 | 14.65 | 10.19 | 13.07 | 5.88 | 12.34 | 7.14 | 10.32 | 8.39 |
| average change | 12.12 | 9.26 | 12.35 | 10.56 | 11.53 | 8.13 | 11.97 | 7.55 | 11.37 | 8.31 |
| ↑: percentage of participants who improved from one time point to the next; ↓: percentage of participants who declined from one time point to the next; improvement/decline is defined by respective succeeding T-score being outside of each child’s/adolescent’s individual confidence interval of the prior measurement point | | | | | | | | | | |

| **Table S4:** Range of difference in T-score by domain per subgroup (decline, improvement) across measurement points | | | | | | | | | | | |
| --- | --- | --- | --- | --- | --- | --- | --- | --- | --- | --- | --- |
| Dimension | Physical wellbeing | | Psychological wellbeing | | Parent relations | | Social support & friends | | School wellbeing | | |
| Time point | Diff range ↑ | Diff range ↓ | Diff range ↑ | Diff range ↓ | Diff range ↑ | Diff range ↓ | Diff range ↑ | Diff range ↓ | Diff range ↑ | Diff range ↓ |  |
| T1 to T2 | 9.51 – 20.56 | -24.25 – -9.02 | 7.61 – 24.51 | -25.61 –-8.19 | 7.37 – 20.67 | -26.98 – -7.62 | 6.26 – 23.61 | -24.34 –-6.65 | 6.22 – 24.81 | -20.83 – -7.28 |  |
| T2 to T3 | 7.15 – 33.12 | -28.22 –-8.69 | 6.89 – 28.10 | -22.74 – -7.28 | 6.72 – 30.61 | -20.67 –-6.85 | 7.01 – 22.04 | -22.73 –-6.77 | 7.13 – 12.48 | -19.20 – -7.47 |  |
| T3 to T4 | 8.47 – 31.81 | -20.24 –-10.99 | 7.68 –24.89 | -20.86 –-6.68 | 7.44 – 22.76 | -15.83– -6.35 | 7.43 – 22.05 | -26.89 – -6.49 | 6.28 – 24.26 | -14.40 – -6.45 |  |
| T4 to T5 | 7.46 – 33.27 | -31.82 –-7.53 | 7.08 – 23.55 | -29.07 –-8.98 | 7.46 – 18.18 | -24.10 –-8.60 | 7.97 – 16.72 | -26.68 – -7.70 | 5.91 – 21.93 | -16.63 – -7.33 |  |
| T5 to T6 | 8.19 – 31.82 | -27.03 –-10.47 | 6.73 – 36.78 | -20.46 – 6.51 | 6.16 – 26.90 | -21.33 –-7.18 | 6.62 – 26.68 | -18.35 – -6.87 | 6.27 – 24.17 | -22.04 – -9.33 |  |
| T6 to T7 | 7.88 – 51.63 | -21.03 –-8.29 | 6.73 – 26.76 | -27.38 – -9.75 | 7.39 – 20. 67 | -23.42 – -8.90 | 8.17 – 23.86 | -18.05 – -8.58 | 6.57 – 31.61 | -25.80 – -7.12 |  |
| Diff range ↑: range of improvement of participants from one time point to the next; Diff range ↓: range of decline of participants from one time point to the next; improvement/decline is defined by respective succeeding T-score being outside of each child’s/adolescent’s individual confidence interval of the prior measurement point | | | | | | | | | | | |
