# Supplementary figures and images for: Trajectories of Health-Related Quality of Life and HbA1c Values of Children and Adolescents With Diabetes Mellitus Type 1 Over 6 Months: A Longitudinal Observational Study
Source: Front Pediatr. 2020 Jan 21;7:566. doi: 10.3389/fped.2019.00566 (PMC6986264; doi:10.3389/fped.2019.00566)

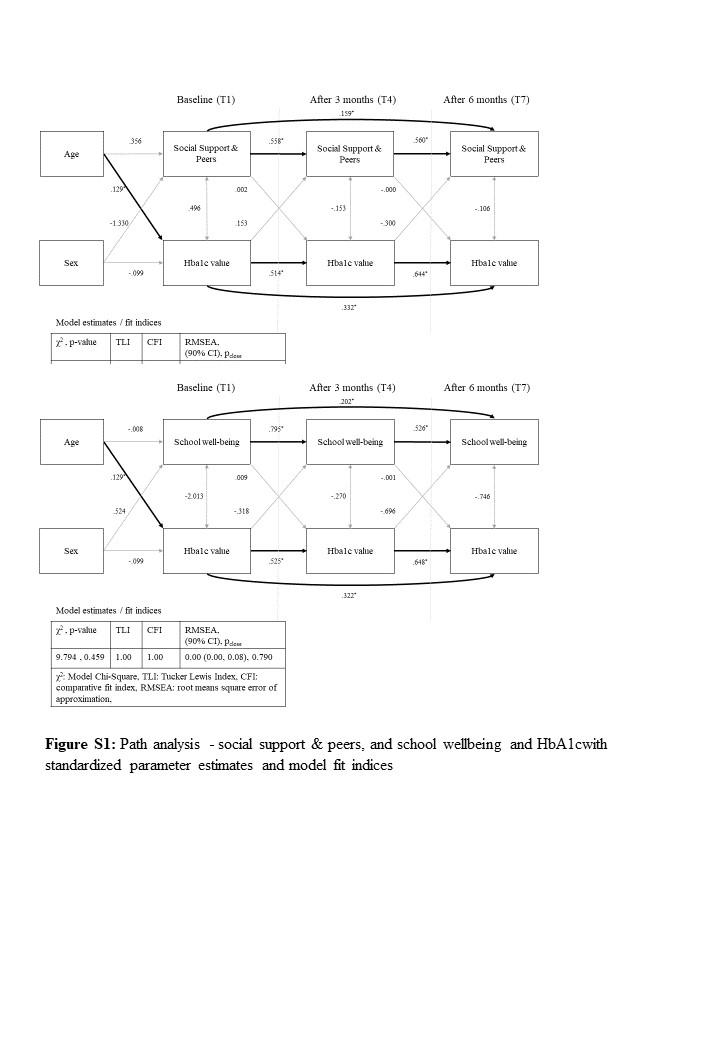

Supplement: Supplementary file 2 [file Image_1.jpg]
